# Sexual attitudes and associated factors of risky sexual behaviors among university students

**DOI:** 10.1002/brb3.2698

**Published:** 2022-07-08

**Authors:** Shayesteh Jahanfar, Zahra Pashaei

**Affiliations:** ^1^ MPH Program, Department of Public Health and Community Medicine Tufts University School of Medicine Boston Massachusetts USA; ^2^ Iranian Research Centre for HIV/AIDS (IRCHA) Tehran University of Medical Sciences Tehran Iran

**Keywords:** attitude, sexual behavior, university students

## Abstract

**Introduction:**

Risky sexual behaviors. Adequate awareness of reproductive health in young people is important because they are in the early years of fertility, and engaging in risky sexual behaviors is more probable. This study aimed to evaluate the sexual attitude and associated factors of risky sexual behaviors among girls and boys and the difference in sexual norms by gender among university students.

**Methods:**

A university‐based study with a cross‐sectional survey was conducted for 9 months in 2019. A total of 800 university students were studied by a random sampling technique using a self‐administered structured questionnaire derived from the World Health Organization illustrative questionnaire to assess sexual attitudes among adolescents and young adults.

**Results:**

Most of the respondents were female and single. Girls were more religious, more frequently visited the cinemas and were more likely to discuss sex matters with family members than boys. There was no significant difference in the total score of sexual attitudes in girls and boys, and both genders had negative attitudes toward risky sexual behavior (42/72). Those who go to parties, bars, or movies are more likely to have risky sexual behavior. Being a religious person is an essential predictor of having less risky behavior (RR = 2.02, 95% CI = [0.96, 3.41]).

**Conclusion:**

Being religious was a protective factor for engaging in risky sexual behavior. More interventions in schools and universities need to educate students to enhance awareness about the consequences of risky sexual behavior and reduce its rate.

## Graphical abstract

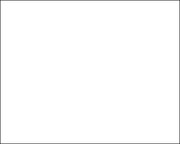

## 1 INTRODUCTION

Risky sexual behaviors (RSB), because of its adverse health consequences, are a serious problem in the health care system. According to the Centers for Disease Control and Prevention (CDC), RSB refers to sexual behaviors that lead to sexually transmitted infections and unintended pregnancies (Brener et al., 2004). The literature suggests the following behaviors as risky: having several partners, having unprotected sex or without contraceptive methods, anal intercourse, initiation of first sex at an early age, having sex impressed by alcohol use, and paid sex (Silas, 2013). Social norms about gender influence the sexual behavior of women and men (Jenkins, 2000). Gender norms refer to ideas or rules about how each gender should behave; they are informal rules adjusting social relationships and actions. These laws are not based on biology but are determined by society's culture, religion, social structure, and economic factors (Morris et al., 2015). Attitudes about traditional gender norms make women more vulnerable than men and increase violence against women (Sommer et al., 2018).

Adequate awareness of reproductive health in young people is important because they are in the early years of fertility and are more likely to engage in high‐risk sexual behaviors (Bollido & Cayabo, 2020). The youth population (age 15–24 years) in the United States in 2018 was reported to be approximately 43 million, with approximately 22 million being between 20 and 24 years of age (10.67 million were female and 11.2 million male), which comprises nearly 13.1% of the total population (Statista, 2019).

Lifelong sex education can extend beyond one generation and reduce the risks of unsafe sexual behavior, such as unintended pregnancies. Limited sexual and reproductive health literacy is one of the main causes of unintended pregnancies (Carstairs et al., 2018). According to reports, approximately 21 million girls aged 15–19 become pregnant every year in developing countries, of which approximately 9.5 million are unwanted (Darroch et al., 2016). In 2011, the highest unintended pregnancy rate in the United States of America was among women aged 20–24 (Finer & Zolna, 2016). It is estimated that if all female adolescents have no plan for pregnancy using any modern contraceptive methods, unintended pregnancies will decrease by two thirds (Darroch et al., 2017). In 2014, more than half of all US abortion patients were in their 20s; patients aged 18–19 and 20–24 years obtained 8% and 34% of all abortions, respectively (Jerman et al., 2016). Education has an important role in preventing unintended pregnancies (WHO, 2011). Moreover, adequate knowledge and appropriate sexual attitudes prevent the negative consequences of RSB, including HIV and other sexually transmitted diseases in youth (Elden et al., 2019).

Evidence showed that reproductive and sex education in school improves awareness about safe sexual behaviors and risk reduction strategies, which had a great impact on the awareness and attitude of young people, especially individuals who have never talked to family or experts about sexual issues and have insufficient and sometimes wrong information (Bergström et al., 2018).

There are vivid findings of the positive association of religious belief and practice and a lower prevalence of RSB (Rosmarin & Pirutinsky, 2019). In similar studies, it was stated that adolescents and young adults who implicated religion in their lives had lower rates of RSB (Smith, 2015).

There has been an association between the early onset of sexual activity and risky behaviors such as smoking cigarettes and using alcohol (Schmid et al., 2007). According to a report by the CDCC, American males smoke cigarettes more than females, and age groups of 18–24 years, including 7.8% of smokers (CDC, 2018). Studies have shown that adolescents who drank alcohol were more likely to have started sexual activity and have RSB (Decat et al., 2015). Smoking cigarettes is an associated risk factor in adolescents and young adults initiating sexual intercourse (Schmid et al., 2007).

In some cultures, one of the desired ways in youth to start an intimate relationship is going to the cinema to watch movies. Because of a lack of awareness about sexual health and undeveloped cognitive skills to analyze the future outcome of risky behaviors in youth, starting intimate relationships may lead to RSB (Gruber & Grube, 2000). Additionally, there have been concerns about the effect of media exposure on “adolescents’ high‐risk behavior and early sex initiation.” It has been shown that “adolescents’ movie exposure influenced later sexual behavior and even sex initiation.” Apparently, among the media, movies have the greatest impact on “adolescents’ beliefs and venture to perform risky behaviors” (Bleakley et al., 2009). Few studies have been conducted about how going to cinema with an opposite gender affects adolescents’ beliefs about having high‐risk sexual behavior and whether necessarily causes to start any kind of sex practice with the companion or not; they may have a close link that it is not easy to explain.

Establishing effective communication between parents and adolescents and their intervention in providing skills and information about healthy sexual behavior prevent the occurrence of high‐risk sexual behaviors (Jones & Jerman, 2017). A recent review found that communication of adolescents with family members and talking about sexual issues had a protective association with RSB (Mmari & Sabherwal, 2013). The fact that youths and young people are influenced by their family, peer groups, and social media shows the importance of multidisciplinary education in the home, school, and mass media, which affects attitudes, knowledge, skills, and norms (Pilgrim & Blum, 2012).

There are some concerns about the beginning of the sexual life of young people; sexual experiences can commence for both genders after a young‐life stable or unstable relationship, instead of an explorative phase that young people could learn safe and right sexual behavior by discovering and building adulthood sexual life gradually. In many cases, the first sexual experiences determine a person's future sex life, and some mistakes and high‐risk sexual relationships are hardly compensable and sometimes irreparable. Examining the prevailing attitudes of young people about sexual behaviors and finding effective factors preventing high‐risk sexual behaviors can help in planning to maintain the sexual health of these people. Furthermore, few studies have explored factors that influence attitudes about sexual behavior and the initiation of sexual activity in young people. In this background, we had the opportunity to enhance our knowledge of the sexual attitude of young people studying in university, such as high‐risk behaviors, sexually transmitted diseases, use of contraception, virginity, sexuality and peers, sexual harassment, sexual satisfaction, and gender norms. The present study aims to provide a new empirical proof of this objective.

## 2 METHODS

### 2.1 Study design

It was a cross‐sectional survey conducted for 9 months in 2019. This study used the World Health Organization (WHO) questionnaire, and several other aspects of the study are discussed elsewhere.

### 2.2 Study population

The inclusion sampling criteria were university students willing to participate in the study. In contrast, the exclusion criterion was students from the Health Science School or those who refused to participate in this study.

### 2.3 Sampling and sample size

A pamphlet with study information was distributed through the departments, ‘‘students’’ housing, and ‘‘students’’ clubs. A list of students’ names and email addresses was obtained from the Registrar's Office. This study used a random sampling technique to send email invitations to students. students in three rounds to obtain an adequate sample size.

The calculated sample size (*n* = 800) was estimated using the prevalence of “being in a romantic relationship” (Lenhart, 2015) of 19%, with an expected increase of 25%, 1.96 *Z* value, 5% precision, 90% power and 20% nonrespondent rate.

### 2.4 Dependent and independent variables used in the survey

We used sociodemographic variables (age, education, work, income, religion, and relationship status) and attending bars/parties, going to movies, drinking alcohol, smoking, and discussing sex matters with family members as confounders. Gender was considered the independent variable, and the total score of RSB was the dependent variable.

### 2.5 Ethical considerations

We first sent an email to participants. In that invitation letter, the aim of the study was explained. Students were asked to spend 20 min filling up the questionnaire. Students were assured that the data would be confidential, as no identification was collected from the students. The risks and benefits of participation in the study were explained to the participants. Students received an email invitation with a link to open the consent form. They signed the consent form before entering the questionnaire. General publications and reports from this study were sent to students for general education purposes. Students who participated in the study were eligible to receive a coupon for Pizza. Students were free to refuse participation or discontinuation of the study at any time point. The Ethics Committee of the Institutional Review Board (IRB) has approved the protocol of the study (IRB: 1031916‐4).

### 2.6 Study questions

The following points should be considered for study questions:

1. The total score of attitudes toward sexuality and norms.
2. Differences between boys and girls in terms of attitude toward sexual norms.

### 2.7 Tool

We used a self‐administered structured questionnaire derived from the WHO illustrative questionnaire (Roger Ingham, n.d.). The questionnaires were sent through Qualtrics software to ensure the anonymity of the responses necessary because of the sensitive nature of the study.

The questionnaire was adopted by the researchers and modified to reflect cultural sensitivities. The change was minor, and no language translation was necessary. Content validity was ensured by sending the questionnaire to 10 students and two faculty members. The data from these 10 students were excluded from the analysis. The main questionnaire assessed students' knowledge, attitudes, and practices about sexual health. The current paper focuses on gender differences in RSB.

A total score of sexual behavior was computed by adding Q1 to Q24 as follows. The total score of sexual attitudes was calculated by adding the following questions: Q1. “I believe it's all right for unmarried boys and girls to have dates” (agree = 1, don't know/not sure = 2, disagree = 3), Q2. “I believe it's all right for boys and girls to kiss, hug and touch each other” (agree = 1, don't know/not sure = 2, disagree = 3), Q3. “I believe there is nothing wrong with unmarried boys and girls having sexual intercourse if they love each other” (agree = 1, don't know/not sure = 2, disagree = 3), Q4. “I think that sometimes a person has to force a partner to have sex if he/she loves her/him” (agree = 3, don't know/not sure = 2, disagree = 1), Q5. “A boy/girl will not respect a girl/boy who agrees to have sex with him/her” (agree = 1, don't know/not sure = 2, disagree = 3), Q6. “Most girls who have sex before marriage regret it afterward” (agree = 3, don't know/not sure = 2, disagree = 1), Q7. “Most boys who have sex before marriage regret it afterward” (agree = 3, don't know/not sure = 2, disagree = 1), Q8. “A boy and a girl should have sex before they become engaged to see whether they are compatible” (agree = 1, don't know/not sure = 2, disagree = 3), Q9. “I believe that girls should remain virgins until they marry” (agree = 1, don't know/not sure = 2, disagree = 3), Q10. “I believe that boys should remain virgins until they marry” (agree = 1, don't know/not sure = 2, disagree = 3), Q11. “It is sometimes justifiable for a boy/girl to hit his girlfriend/boyfriend” (agree = 3, don't know/not sure = 2, disagree = 1), Q12. “Most of my friends think that one‐night stands are OK” (agree = 1, don't know/not sure = 2, disagree = 3), Q13. “It's all right for boys and girls to have sex with each other provided that they use methods to stop pregnancy” (agree = 1, don't know/not sure = 2, disagree = 3), Q14. “Most of my friends who have sex with someone use condoms regularly” (agree = 1, don't know/not sure = 2, disagree = 3), Q15. “I am confident that I can insist on condom use every time I have sex” (agree = 1, don't know/not sure = 2, disagree = 3), Q16. “I would never contemplate having an abortion myself or for my partner” (agree = 1, don't know/not sure = 2, disagree = 3), Q17. “It is mainly the woman's responsibility to ensure that contraception is used regularly” (agree = 3, don't know/not sure = 2, disagree = 1), Q18. “I think that you should be in love with someone before having sex with them” (agree = 1, don't know/not sure = 2, disagree = 3), Q19. “I feel that I know how to use a condom properly” (agree = 1, don't know/not sure = 2, disagree = 3), Q20. “Most of my friends would never contemplate having an abortion for themselves or their partner” (agree = 1, don't know/not sure = 2, disagree = 3), Q21. “Men need sex more frequently than do women” (agree = 3, don't know/not sure = 2, disagree = 1), Q22. “Most of my friends believe that you should be in love before you have sex with someone” (agree = 1, don't know/not sure = 2, disagree = 3), Q23. “I would refuse to have sex with someone who is not prepared to use a condom” (agree = 1, don't know/not sure = 2, disagree = 3), Q24. “One‐night stands are OK” (agree = 3, don't know/not sure = 2, disagree = 1).

### 2.8 Data analysis

Statistical analysis of these data was performed using IBM SPSS Statistics, version 26.0, released in 2019 (IBM Corp., Armonk, NY, USA). Descriptive, bivariate, and linear regression analyses were performed to analyze these data. Regression analysis was conducted to find the predictive relationship between the sexual behavior of the students and their sociodemographic characteristics. As the total score of sexual behavior is a continuous variable, the relative risk (RR) and the 95% confidence intervals (CI) were calculated.

## 3 RESULTS

### 3.1 Descriptive analysis

The demographic characteristics, educational background, and relationship status are summarized in Table 1. Most of the respondents were female, 79.3% (636), and 20.7% (166) were male. The distribution of age in the two sexes was the same. The majority of students were undergraduates (549, 68.2%). Most of them believed in one type of religion (471, 60.7%) and were single (601, 95.4%).

**TABLE 1:** Sociodemographic characteristics (*n* = 804)

Variables	N (%)
Age (mean ± SD)	23.87±7.56
**Gender**	
Male	167 (18.1)
Female	636 (68.8)
**Education**	
Undergraduate	549 (68.2)
Graduate	256 (31.8
**Work**	
Yes	520 (68.8)
No	236 (31.2)
**Income**	
$1000 or less	721 (78.0)
More than $1000	203 (22.0)
**Are you a religious person?**	
Yes	471 (60.7)
No	305 (39.3)
**Relationship status**	
Single	601 (95.4)
No single	29 (4.6)
**Total Score of sexual attitudes**	42.46 (±3.66)

### 3.2 Gender comparison

Differences between girls and boys were found concerning being religious (*p* = .02), as the girls were more religious. Girls more frequently reported going to movies (*p* = .01). On the other hand, more boys (540 out of 665) smoked cigarettes at the time of the survey or earlier (*p* = .01). The difference between girls and boys was statistically significant (*p* = .01) in discussing sex matters with family members, and girls discussed more than boys. There was no significant difference in the total score of sexual attitudes in girls (636) and boys (166), 42.42 ± 3.61 and 42.59 ± 3.87, respectively. The attitude of both genders was at the appropriate level, and they had a negative attitude toward RSBs (42/72) (Table 2).

**TABLE 2:** Comparison of sociodemographic characteristics, sources of information, and risky sexual behavior between male and female students (*n* = 802)

	Male	Female	
	N = 166	N = 636	*p*
Age (mean ± SD)	23.59 ± 6.62	23.95 ± 7.81	.58
**Education**			
Undergraduate	110 (20.1)	437 (79.9)	.55
Graduate	56 (22.0)	199 (78.0)	
**Work**			
Yes	109 (21.0)	409 (79.0)	.63
No	46 (19.5)	190 (80.5)	
**Income**			
$1000 or less	86 (20.1)	342 (79.9)	.60
More than $1000	47 (21.9)	168 (78.1)	
**Are you a religious person?**			
Yes	79 (25.9)	226 (74.1)	.02
No	36 (19.0)	153 (81.0)	
**Relationship status**			
Single	126 (21.0)	475 (79.0)	.17
No single	3 (10.3)	26 (89.7)	
**Do you ever go to bars/parties?**			
Yes	119 (20.0)	476 (80.0)	.30
No	40 (23.7)	129 (76.3)	
**Do you ever go to movies?**			
Yes	140 (19.7)	569 (80.3)	.01
No	20 (39.2)	31 (60.8)	
**Do you ever drink alcohol?**			
Yes	133 (20.2)	527 (79.8)	.12
No	26 (27.1)	70 (72.9)	
**Do you ever smoke cigarettes?**			.01
Yes	33 (34.4)	63 (65.6)	
No	125 (18.8)	540 (81.2)	
**Discuss sex matters with family member**			
Yes	47 (18.0)	214 (82.0)	.01
No	15 (40.5)	22 (59.5)	
**Total score of sexual attitudes** *	42.59 ± 3.87	42.42 ± 3.61	.70

* Mann–Whitney *U* test.

### 3.3 Regression analysis

Bivariate and linear regression analyses assessed the risk ratio of sexual risk behaviors and predictive values (Table 3). Those who were religious had a negative attitude toward RSBs (RR = 1.14, 95% CI = [0.42, 1.86]). Going to bars or parties was associated with RSB (RR = −1.07, 95% CI = [−1.92, −0.23]), so students who go to bars or parties were more likely to engage in RSBs. Similarly, those who smoked cigarettes were more likely to have RSB (RR = −2.13, 95% CI = [−3.19, −1.07]). In linear regression, it was found that being religious is an important interpersonal predictor of having less risky behavior (RR = 2.02, 95% CI = [0.96, 3.41]).

**TABLE 3:** Unadjusted and adjusted risk ratios of risky sexual behavior and predictive values

	Unadjusted RR	Adjusted RR
	(95% CI)	(95% CI)
**Gender**		
Male	0.17 (−0.70, 1.04)	0.05 (−1.55, 1.65)
Female	1	1
**Age (mean ± SD)**	−0.02 (−0.08, 0.04)	0.06 (−0.06, 0.19)
**Education**		
Undergraduate	0.12 (−0.67, 0.92)	0.59 (−1.15, 1.65)
Graduate	1	1
**Work**		
Yes	0.15 (−0.61, 0.91)	−0.09 (−1.72, 1.52)
No	1	1
**Income**		
$1000 or less	−0.04 (−0.89, 0.81)	−1.08 (−2.81, 0.65)
More than $1000	1	1
**Are you a religious person?**		
Yes	1.14 (0.42, 1.86)	2.02 (0.96, 3.41)
No	1	1
**Relationship status**		
No single	0.79 (−1.31, 2.89)	0.82 (−2.37, 4.02)
Single	1	1
**Do you ever go to bars/parties?**		
Yes	−1.07 (−1.92, −0.23)	−0.67 (−2.49, 1.16)
No	1	1
**Do you ever go to movies?**		
Yes	0.45 (−1.04, 1.94)	−0.86 (−4.24, 2.52)
No	1	1
**Do you ever drink alcohol?**		
Yes	−0.86 (−1.88, 0.15)	−1.40 (−3.81, 1.01)
No	1	1
**Do you ever smoke cigarettes?**		
Yes	−2.13 (−3.19, −1.07)	0.04 (−2.24, 2.31)
No	1	1
**Discuss sex matters with family member**		
Yes	0.71 (−1.04, 2.45)	0.84 (−1.49, 3.17)
No		1

## 4 DISCUSSION

Our study highlights the relation of demographic characteristics of participants with RSBs and students’ attitudes toward it. The attitude score of the students participating in the study showed that their attitude level was at an appropriate level, and they had a negative attitude toward RSBs. Few adolescents in our study sample were in a relationship at the time of the survey, and most of them were single.

A comparison of girls and boys found that girls discuss sex matters with family more than boys. The involvement of parents in the sexual education of their children has an undeniable impact on the sexual health of young people. According to studies’ findings, adolescents prefer to talk about sexual issues with mothers, and mothers feel more comfortable talking about sexual matters with girls than boys (Trinh et al., 2009). One possible reason for more mother–daughter sexual talk is girls’ menstruation as a biological marker, which requires definite action and education in the family. Another reason is that daughters’ sexual activity seems dangerous because of the probability of pregnancy (Walker, 2001). Consequently, girls talked more about sexual matters with parents than boys. The findings of a study showed that approximately 33% of both young girls and boys feared discussing sexual matters with family because of less trust and confidentiality of sexual health issues (Sleem & Mekhael, 2016). Girls go to the movies more than boys. In our searches, no study was found about the relationship between going to the cinema and RSB. Additionally, there is a lack of information about the difference in going to the cinema between the two genders. There is a lack of information. Additionally, they were more religious than their male peers. A similar result was reported in a study that examined the impact of sociodemographics and religiosity on RSB (Agardh et al., 2011). The theory of attachment has been proposed as one of the reasons why women are religious, where God assumed a secure base for believers (Flannelly & Galek, 2010). Women possess more positive God images than men, and communicating with God as a secure base haven helps their psychological well‐being (Krejci, 1998). Continuing the discussion of comparing the two genders, boys smoke cigarettes more than girls, which matches the findings of youth risk behavior surveillance performed in the United States (Kann et al., 2016). Related studies suggest that men smoke for different reasons compared to women, which may relate to a combination of physiological, cultural, and behavioral factors, including reinforcing the effect of nicotine, social acceptability, and looking more attractive (Parkinson et al., 2009).

The present study revealed that students who go more frequently to bars or parties were more likely to have RSB. Girls went to parties and bars slightly more than boys (79%, 74.8%). Incompatible with these findings, those who frequently attended nightclubs were more likely to have RSB and even showed a significant association with having sex ever (Perera & Abeysena, 2018).

RSB was higher in respondents who smoked cigarettes at the time of the survey. Boys and girls who smoked cigarettes were more likely to experience high‐risk behaviors. Other studies are concordant with our findings on smoking cigarettes and RSB (Berhan et al., 2013).

There was a protective association between being religious and having RSB. In other words, students who played a bold role in their lives were less likely to have RSB than those who were not. Recent studies provide evidence to support this finding, such that young people who were not religious and did not participate in church religious activities did not adhere to moral principles and safe sexual behavior (Hill et al., 2014). Additionally, three studies performed among undergraduate university students in the United States, Sri Lanka, and Ethiopia revealed similar findings. In the first study, of those 3.168 women and men who contributed to the survey, who had religion as a crucial part of their life, attended church frequently, and had religious, sexual attitudes were not only 27%–54% less likely to have sex but also had fewer sex partners than their counterparts (Haglund & Fehring, 2010). In the second study conducted in Sri Lanka, those who frequently engaged in religious activities and religion was an important part of their lives (1238/1575) were 1.5 times less likely to engage in high‐risk behavior than others (Perera & Abeysena, 2018). In a study conducted recently, students who attended religious institutions regularly had lower odds of RSB than those who never attended (Ware et al., 2018). In contrast to the findings, a study showed that students who had religious beliefs and had less religious behavior were more likely to engage in RSB. A possible explanation for this finding could be that young people want to break free from preuniversity restrictions and engage in sexual practices that they have never had the chance to experience before (Prassel, 2016).

This study has given us a cross‐sectional view in a short period. More comprehensive studies are recommended for detailed review and assessing risk factors in the long term. It was a sensitive and self‐reported study, and participants may have withheld some information due to unacceptable risky behaviors. In our study, the positive response of ‘‘are you religious person’’ was assumed to be religious, and we did not distinguish between religious belief and religious behavior. Future studies should be designed to assess the relation of religion and RSB with precise differentiation between religious belief and religious behavior.

This study has several potential limitations. First, we performed this study on young people who were studying in university, so the generalization of this study's findings to other youths in other areas and out of university is limited. Second, although the anonymity of respondents was preserved during the self‐completion of the questionnaire, there is a possibility of underreporting bias because of the sensitivity of premarital sex in society.

## 5 CONCLUSION

The current study investigated the relationship between potential risk factors and RSB in undergraduate students. Attitudes toward RSB were negative. Those who were religious were associated with less RSB. Schools should provide comprehensive education with an orientation on gender equality and safe sexual behaviors for all students, whether sexually active or inactive, and counsel them to minimize risks when engaging in sexual practice and choose the method of pregnancy prevention that could meet their needs. Talking about gender equality in the long term will reduce gender‐based violence in society by changing minds, attitudes, and, finally, acts of individuals, which will lead to a change in detrimental gender norms. The curriculum of sex education presented in schools and universities must change as norms, values, and practices change to meet the young population's needs. Future studies should investigate how gender norms cause behavioral differences in girls and boys to change. An interventional study should be designed to adjust the gender norms in the minds of students to change gender‐oriented RSBs.

## CONFLICT OF INTEREST

The authors declare that they have no conflicts of interest.

## AUTHOR CONTRIBUTIONS

*Supervisor, project administration, study design, conceptualization, methodology, software, data curation, data analysis*, *review, and editing*: Shayesteh Jahanfar. *Study design, visualization, data collection, data analysis, writing original draft, review, and editing*: Zahra Pashaei.

## FUNDING INFORMATION

None.

## IMPLICATION AND CONTRIBUTION

Risky sexual behaviors in young adults may cause serious health problems and affect their whole lives. In this study, attitudes toward risky sexual behavior were negative. Adolescents who were religious had less risky sexual behavior.

### PEER REVIEW

The peer review history for this article is available at https://publons.com/publon/10.1002/brb3.2698.

## Data Availability

Data sharing is not applicable.
